# Myeloid-derived growth factor ameliorates dextran sodium sulfate-induced colitis by regulating macrophage polarization

**DOI:** 10.1007/s00109-024-02447-3

**Published:** 2024-05-02

**Authors:** Yang Yang, Conghui Zhao, Zi Yang, Conglin Du, Zhichao Chang, Xin Wen, Xiujuan Zhang, Yi liu, Liang Hu, Zhenhua Gao

**Affiliations:** 1 Department of Oral and Maxillofacial & Head and Neck Oncology, Beijing Stomatological Hospital, Capital Medical University, Beijing, 100050 China; 2 Department of Pathology, Beijing Stomatological Hospital, Capital Medical University, Beijing, 100050 China; 3 Department of Endodontics, Beijing Stomatological Hospital, Capital Medical University, Beijing, 100050 China; 4 Salivary Gland Disease Center and Beijing Key Laboratory of Tooth Regeneration and Function Reconstruction, School of Stomatology and Beijing Laboratory of Oral Health, Beijing, 100050 China; 5 Nephrology Department, Zhucheng People’s Hospital, Shandong, 262200 China; 6 Laboratory of Tissue Regeneration and Immunology and Department of Periodontics, Beijing Key Laboratory of Tooth Regeneration and Function Reconstruction, School of Stomatology, Capital Medical University, Beijing, 100050 China; 7 Department of Oral and Maxillofacial Surgery, Beijing Stomatological Hospital, Capital Medical University, Beijing, 100050 China

**Keywords:** Colitis, Inflammatory bowel disease, Myeloid-derived growth factor, Dextran sulfate sodium

## Abstract

**Abstract:**

Inflammatory bowel disease (IBD) is characterized by inflammatory conditions in the gastrointestinal tract. According to reports, IBD prevalence is increasing globally, with heavy economic and physical burdens. Current IBD clinical treatment is limited to pharmacological methods; therefore, new strategies are needed. Myeloid-derived growth factor (MYDGF) secreted by bone marrow-derived mononuclear macrophages has beneficial effects in multiple inflammatory diseases. To this end, the present study aimed to establish an experimental IBD mouse model using dextran sulfate sodium in drinking water. MYDGF significantly alleviated DSS-induced colitis, suppressed lymphocyte infiltration, restored epithelial integrity in mice, and decreased apoptosis in the colon tissue. Moreover, the number of M1 macrophages was decreased and that of M2 macrophages was increased by the action of MYDGF. In MYDGF-treated mice, the NF-κB and MAPK pathways were partially inhibited. Our findings indicate that MYDGF could mitigate DSS-induced mice IBD by reducing inflammation and restoring epithelial integrity through regulation of intestinal macrophage polarization via NF-κB and MAPK pathway inhibition.

**Key messages:**

- MYDGF alleviated DSS-induced acute colitis.
- MYDGF maintains colon epithelial barrier integrity and relieves inflammation.
- MYDGF regulates colon macrophage polarization.
- MYDGF partially inhibited the activation of NF-κB and MAPK pathway.

**Supplementary Information:**

The online version contains supplementary material available at 10.1007/s00109-024-02447-3.

## Introduction

Ulcerative colitis (UC) is an idiopathic inflammatory bowel disease (IBD) that is mainly confined to inflammatory lesions in the colon [1, 2]. Although the pathological mechanism of UC is unclear, the destruction of the epithelial barrier is widely believed to lead to the invasion of the mucosal layer by bacterial antigen, which results in activation of the mucosal immune system [3]. Excess inflammatory cytokine and immune cell activation exacerbate the inflammatory environment in UC, ultimately triggering an immune response in the colon [4, 5].

The prevalence of IBD, including UC and Crohn's disease (CD; an IBD subtype), is increasing globally, particularly in developing countries [6, 7]. Current treatment strategies for UC include the use of corticosteroids, anti-inflammatory drugs, and immunosuppressants; however, existing treatments have not been satisfactory [8]. Therefore, novel therapeutic strategies and biologics are required to alleviate UC.

Myeloid-derived growth factor (MYDGF) is a protein secreted endogenously by bone marrow-derived monocytes and macrophages, which is encoded by an open reading frame on chromosome 19 (C19orf10) [9]. Based on its primary amino acid sequence, MYDGF does not belong to any known growth factor or cytokine family [10]. MYDGF has a good therapeutic effect on ischemia-reperfusion injury and can repair myocardial infarction and angiogenesis [9, 11]. Moreover, MYDGF regulates MAP4K and NF-κB pathways to inhibit inflammation and endothelial damage [12]. The present study shows that MYDGF has great therapeutic potential in immune diseases. Considering IBD is an immune-related disease, and macrophages are key players in the intestinal immune system that regulate intestinal homeostasis [13, 14], the authors hypothesized that MYDGF could alleviate IBD by modulating the immune microenvironment.

Dextran sulfate sodium (DSS) is widely used for simulating UC [15]. DSS can disrupt the integrity of the intestinal epithelium, leading to a localized inflammatory response [16–18]. In the present study, mice were fed with 3% DSS water to induce acute colitis. The role of MYDGF in DSS-induced colitis was subsequently evaluated by injecting MYDGF into the caudal vein. MYDGF significantly alleviates the symptoms of DSS-induced colitis. Considering the key role of macrophage polarization in the immune system [19], the authors demonstrated that MYDGF regulates colonic macrophage polarization via inhibition of the NF-κB and MAPK pathways to alleviate DSS-induced colitis.

## Results

### MYDGF alleviated DSS-induced acute colitis

**Fig. 1:**
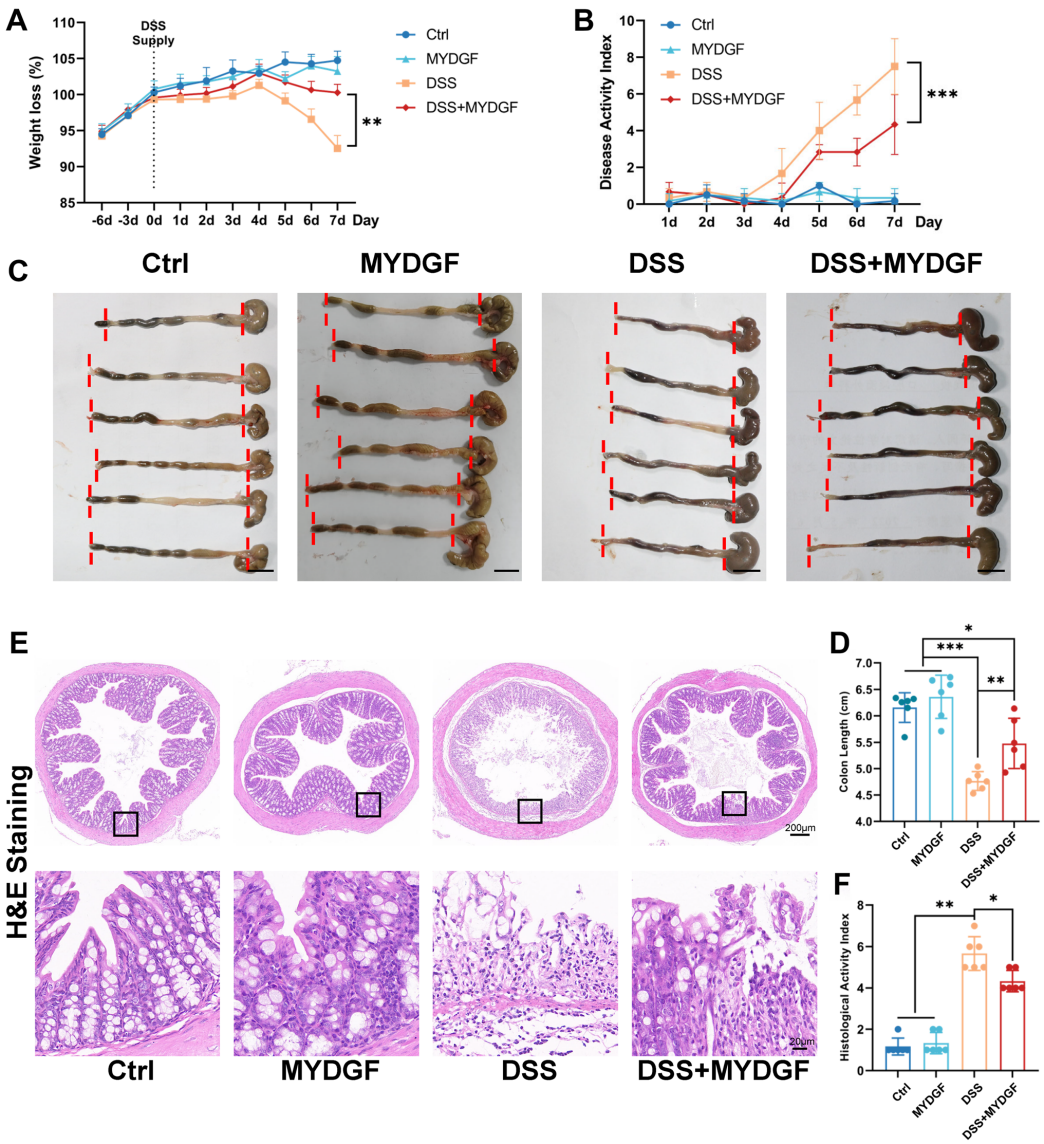
MYDGF alleviated DSS-induced acute colitis. **A** Four groups of body weight loss daily (Initial body weight as 100%); **B** Four groups of DAI daily; **C** The colons of four groups (the red line shows the length of the colon, scale bar: 1 cm); **D** Colon length of the four groups; **E** H&E staining and **F** HAI score in response to DSS and MYDGF (The bottom image in H&E staining is the field of view in the enlarged black frame of the upper image). *p < 0.05, **p < 0.01, ***p < 0.001. n = 6, two-way analysis of variance (ANOVA) was used to compare the differences among two groups with multiple columns, One-way analysis of variance (ANOVA) was used to compare the differences among multiple groups

After seven days of 3% DSS supplementation in drinking water, the mice developed acute colitis symptoms. Both the DSS and DSS+MYDGF groups had different degrees of liquid and bloody stools (Supplementary Fig. 1A). Throughout the study, mice were weighed, and the values recorded daily (Supplementary Fig. 1B). The percentage weight loss and disease activity index (DAI) were calculated daily (Fig. 1A, B). Drinking of DSS water caused significant weight loss and increased DAI on day 5. Compared with that in the DSS group, the degree of weight loss was lower in the DSS+MYDGF group. On day 7, body weight in the DSS+MYDGF group was significantly higher than that in the DSS group. Compared with that in the DSS+MYDGF group, DAI was significantly higher in the DSS group.

Whole blood samples were collected after anesthesia for routine blood examination. Drinking of DSS water significantly reduced the number of red blood cells (RBC), hematocrit (HCT), and hemoglobin (HGB), and increased the number of white blood cells (WBC) and lymphocytes (LYMPH) in mice (Table 1). Single injection of MYDGF into the tail vein had no effect on all blood indexes. Compared with the DSS group, WBC, LYMPH, and lymphocyte percentage (LYMPH%) were reduced significantly in the DSS+MYDGF group. However, MYDGF did not restore RBC, HCT, and HGB reduced by DSS drinking. In addition, the number of platelets (PLT) decreased in the DSS group; however, the decrease was not significant. Besides, monocyte counts (MONO), monocyte percentage (MONO%), and other indicators were measured (Supplementary Table S1).

**Table 1:** Blood routine examination

	**Ctrl**	**MYDGF**	**DSS**	**DSS+MYDGF**
WBC	4.89 ± 1.78	4.59 ± 1.48	8.49 ± 1.39^a,b^	5.19 ± 1.47^c^
RBC	8.88 ± 0.60	8.82 ± 0.32	6.79 ± 1.98^a,b^	6.45 ± 1.92^a,b^
HCT	42.00 ± 2.68	41.50 ± 1.36	33.18 ± 8.18^a,b^	32.90 ± 7.48^a,b^
HGB	134.67 ± 9.73	132.17 ± 6.08	104.17 ± 29.77^a,b^	98.83 ± 28.58^a,b^
PLT	928.50 ± 81.13	934.33 ± 91.97	818.00 ± 168.3	917.17 ± 80.18
LYMPH	4.40 ± 1.66	3.68 ± 1.08	6.63 ± 0.69^a,b^	4.19 ± 1.53^c^
LYMPH%	89.23 ± 3.96	88.16 ± 7.40	89.96 ± 1.61	80.18 ± 9.16^c^

One-way analysis of variance (ANOVA) was used to compare the differences among multiple groups

n = 5–6

^a^Compared with Ctrl group, p < 0.05

^b^Compared with MYDGF group, p < 0.05

^c^Compared with DSS group, p < 0.05

The mice were euthanized humanely, after which their colons were collected, and their lengths were measured (Fig. 1C, D). DSS treatment reduced colon length, whereas MYDGF partially restored the lost colon length. Pathological evaluation was performed and scored based on the histological activity index (HAI) in hematoxylin and eosin (H&E) stains (Fig. 1E–F). MYDGF partially alleviated lymphocyte infiltration and epithelial integrity damage caused by DSS treatment.

### MYDGF alleviated DSS-induced apoptosis in colon

**Fig. 2:**
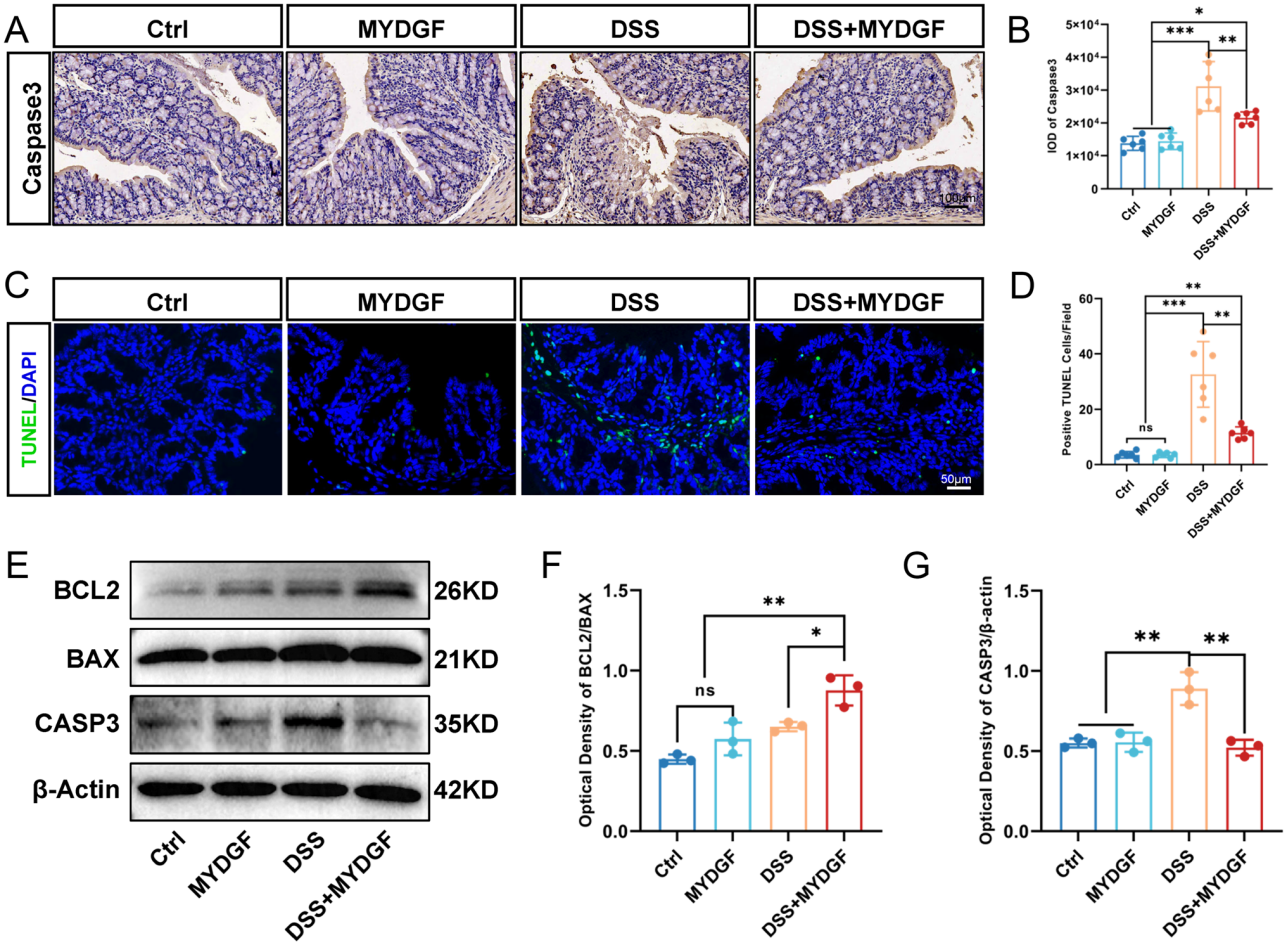
MYDGF alleviated DSS-induced apoptosis. **A**,** B** The expression of Caspase 3 was examined by immunohistochemistry staining and quantified, scale bar: 100 μm; **C**, **D** TUNEL was examined by immunofluorescent staining (greeen) and quantified, scale bar: 50 μm; **E**–**G** The expression and quantitative analysis of BAX, BCL-2 and Caspase-3 in four groups in western blotting. *p < 0.05, **p < 0.01 and ***p < 0.001. n = 3–6, One-way analysis of variance (ANOVA) was used to compare the differences among multiple groups and unpaired non-parametric t-test was used to compare two groups for western blot

Apoptosis is the major form of cell death caused by DSS-induced colitis. Immunohistochemistry (IHC) for Caspase-3 was used to observe apoptosis. Semi-quantitative analysis of Caspase-3 expression showed that consumption of MYDGF alone did not cause colon damage. MYDGF partially prevented DSS-induced increase in Caspase-3 expression (Fig. 2A, B). Terminal deoxynucleotidyl transferase (TdT) dUTP nick-end labeling (TUNEL) assay was performed to detect DNA degradation in the apoptosis response. Analysis of TUNEL-positive cells revealed that MYDGF reduced DSS-induced colon tissue apoptosis (Fig. 2C, D). The expression levels of the pro-apoptotic protein BAX, Caspase-3, and anti-apoptotic proteins BCL2 were detected by western blotting and semi-quantitative analysis, respectively (Fig. 2E–G). MYDGF promoted BCL2 expression and decreased Caspase-3 expression. These findings indicate that MYDGF alleviates DSS-induced colitis in mice by reducing tissue apoptosis.

### MYDGF maintains colon epithelial barrier integrity and relieves inflammation

**Fig. 3:**
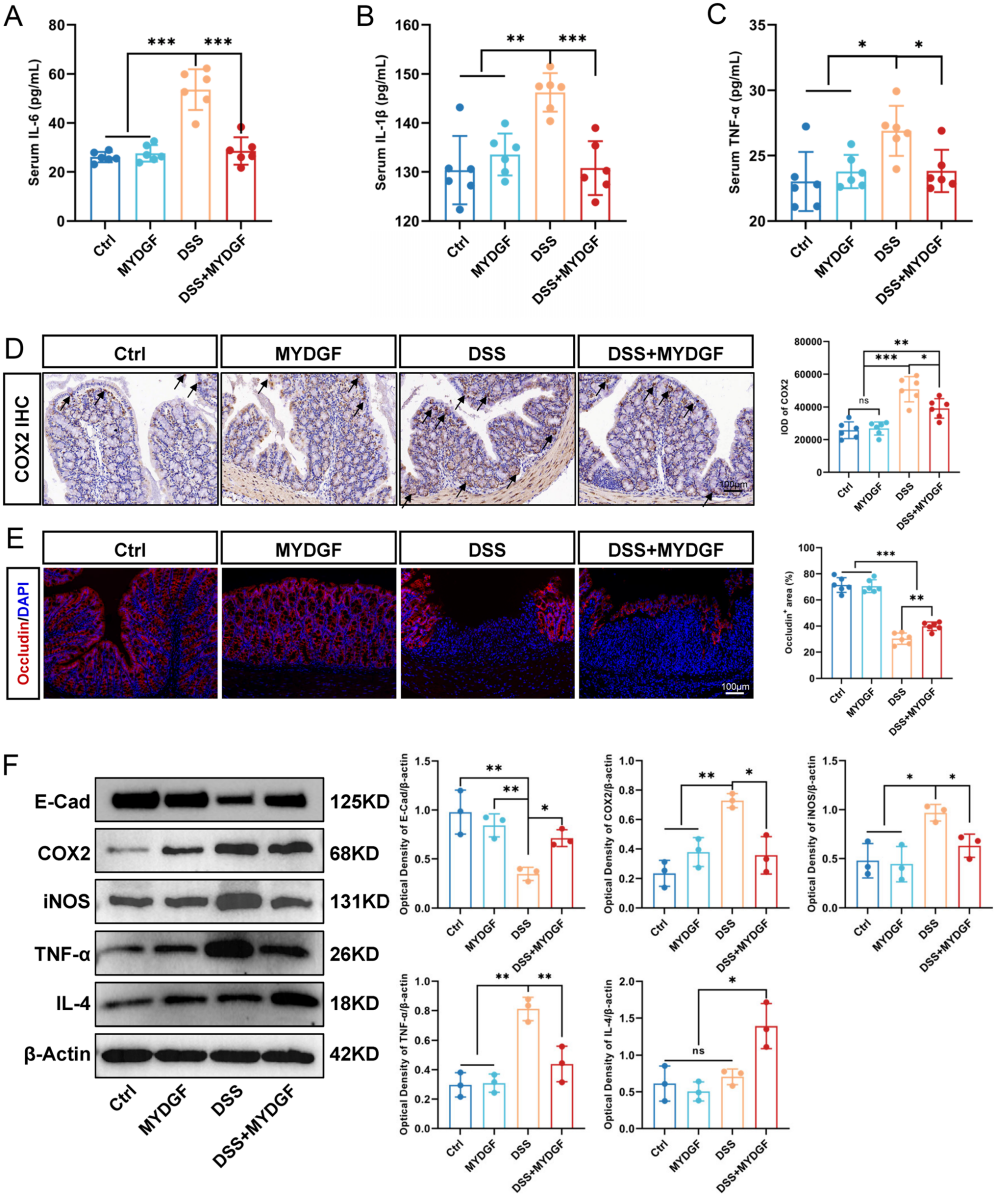
MYDGF maintains colon epithelial barrier integrity and relieves inflammation. **A**–**C** The serum concentration of IL-6, TNF-α, and IL-1β were measured by ELISA; **D** The expression of COX2 was examined by immunohistochemistry and quantified (The black arrow points to the COX2^+^ cell), scale bar: 100 μm; **E** The expression of Occludin (red) was examined by immunofluorescent and quantified, scale bar: 100 μm; **F** The expression and quantitative analysis of E-cad, COX2, iNOS, TNF-a, and IL-4 in western blotting. *p < 0.05, **p < 0.01 and ***p < 0.001. n = 3–6, One-way analysis of variance (ANOVA) was used to compare the differences among multiple groups and unpaired non-parametric t-test was used to compare two groups for western blot

The serum contents of IL-6, TNF-α, and IL-1β were measured through enzyme-linked immunosorbent assay (ELISA) (Fig. 3A–C). MYDGF decreased the secretion of the inflammatory cytokines. Cyclooxygenase 2 (COX2) is expressed in large quantities when cells are stimulated by inflammation cytokines [20]. The expression of COX2 (Fig. 3D) was detected and analyzed. DSS water drinking enhanced the COX2 expression significantly, whereas MYDGF reduced COX2 expression. Occludin and E-Cadherin (E-Cad) are intercellular cell adhesion molecules that maintain epithelial integrity [21]. Occludin was detected and analyzed to assess the integrity of the colonic epithelium (Fig. 3E). The IHC of E-Cad was detected and analyzed to further confirm the integrity of the colonic epithelium (Supplementary Fig. 2). MYDGF can enhance the occludin-positive area, suggesting that MYDGF can maintain colon epithelial integrity. In addition, western blotting was used to detect and analyze E-Cad, COX2, inducible nitric oxide synthase (iNOS), and tumor necrosis factor-α (TNF-α) expression (Fig. 3F), which showed a trend similar to the above results. Additionally, the anti-inflammatory factor interleukin 4 (IL-4) was detected. MYDGF enhanced the expression of IL-4 significantly. Overall, these results imply that MYDGF maintained the integrity of the colonic epithelial barrier and relieved the colonic inflammatory response induced by DSS treatment.

### MYDGF regulates colon macrophage polarization

**Fig. 4:**
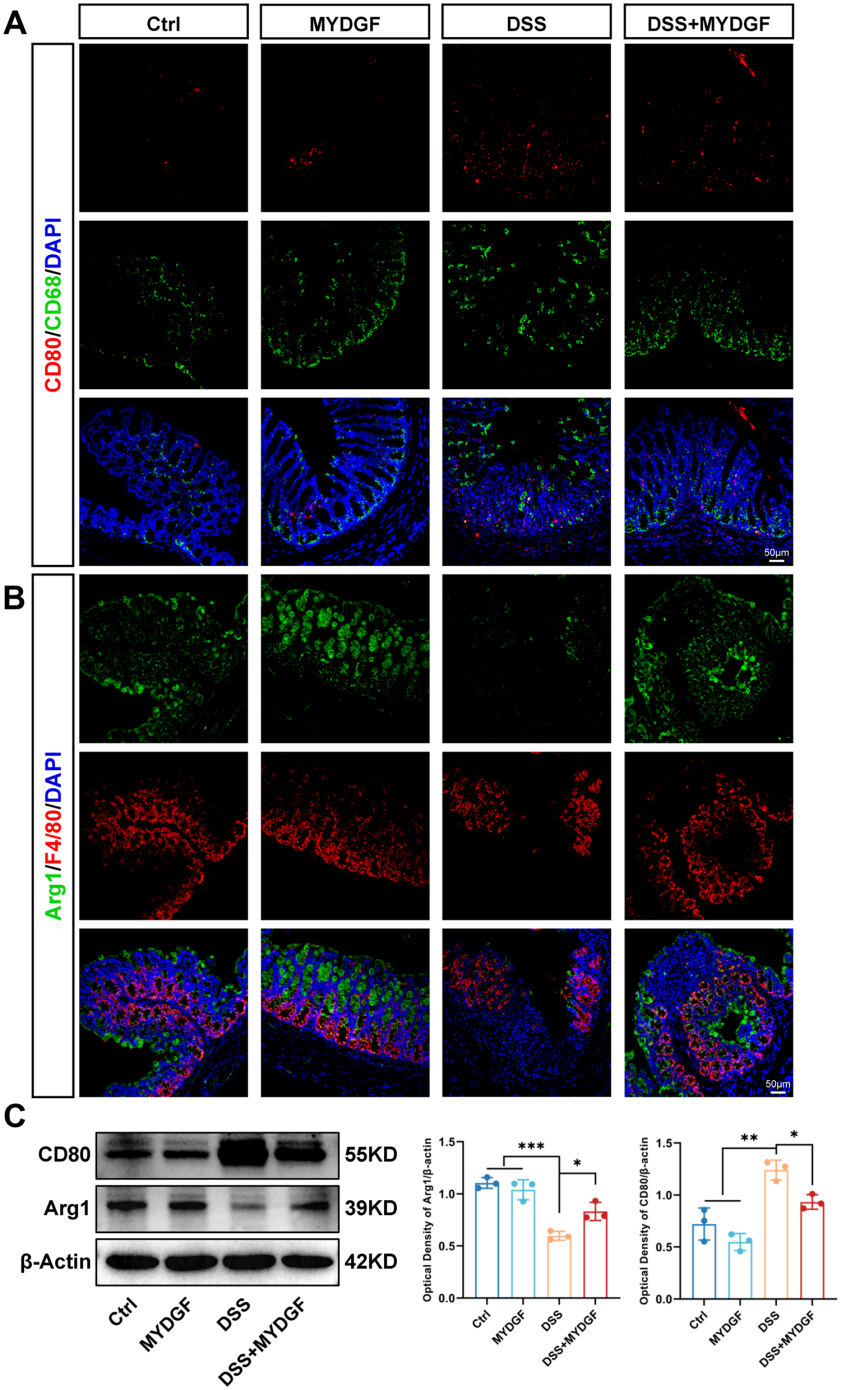
MYDGF regulates colon macrophage polarization. **A** The effect of MYDGF on M1 macrophage polarization. Sections of colon tissues were immunostained with DAPI (blue), CD68 (green) and CD80 (red), scale bar: 50 μm; **B** The effect of MYDGF on M2 macrophage polarization. Sections of colon tissues were immunostained with DAPI (blue), F4/80 (red) and Arg1 (green), scale bar: 50 μm; **C** The expression and quantitative analysis of CD80 and Arg1 in western blotting. *p < 0.05, **p < 0.01 and ***p < 0.001. n = 3. One-way analysis of variance (ANOVA) was used to compare the differences among multiple groups

Macrophage activation is one of the main inflammatory responses. We considered the close relationship between the NF-κB and MAPK signaling pathways and M1/M2 macrophage polarization [22, 23]. The macrophage marker CD68 (green) and M1 marker CD80 (red) were used to observe the M1 polarization (Fig. 4A). DSS water drinking increased the polarization of M1, and MYDGF could decrease the polarization of M1. To distinguish it from the previous results, macrophage marker F4/80 was labeled with red fluorescence, and M2 marker arginase-1 (Arg1) (green) was used to observe the M2 polarization (Fig. 4B). DSS water drinking decreased the polarization of M2, and MYDGF increased the polarization of M2 more than the DSS group. The western blotting was used to detect and analyze CD80 and Arg1 expression (Fig. 4C), MYDGF could decrease the polarization of M1 and increased the polarization of M2. Based on the findings, we believe that MYDGF decreases M1 and increases M2 polarization in macrophages.

### MYDGF partially inhibited the activation of NF-κB and MAPK pathways

**Fig. 5:**
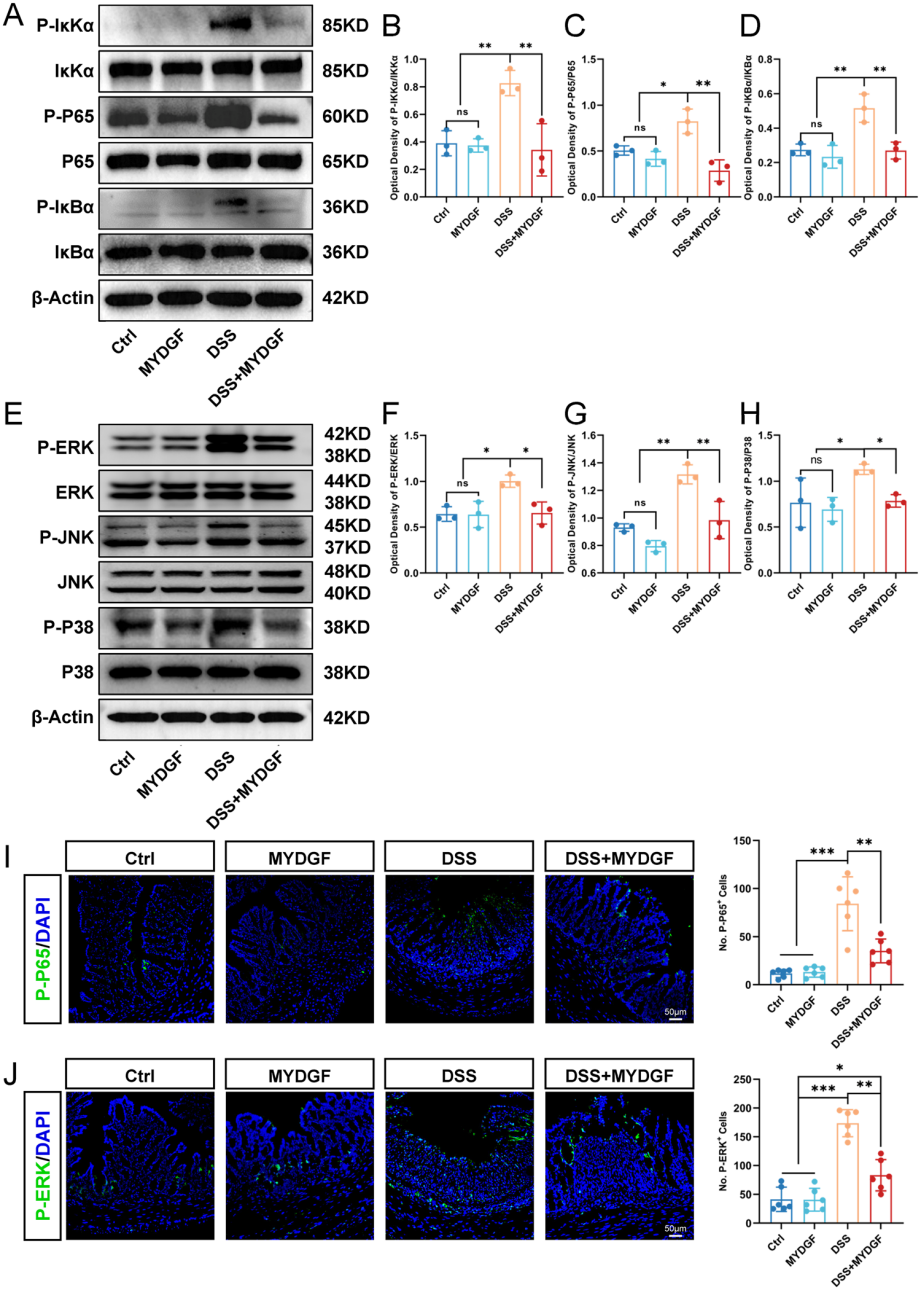
MYDGF partially inhibited the activation of NF-κB and MAPK pathway. **A** The phosphorylation of IKKα, P65, and IκBα in NF-κB pathway by western blotting; **B**–**D** The quantitative analysis of phosphorylation of IKKα, P65, and IκBα; **E** The phosphorylation of ERK, JNK, and P38 in MAPK pathway by western blotting; **F**–**H** The quantitative analysis of phosphorylation of ERK, JNK, and P38. **I** The expression of P-P65 (green) was examined by immunofluorescent and quantified, scale bar: 50 μm; **J** The expression of P-ERK (green) was examined by immunofluorescent and quantified, scale bar: 50 μm. *p < 0.05, **p < 0.01 and ***p < 0.001. n = 3–6, One-way analysis of variance (ANOVA) was used to compare the differences among multiple groups and unpaired non-parametric t-test was used to compare two groups for western blot

The NF-κB and MAPK signaling pathways are involved in DSS-induced acute colitis [24]. To determine whether MYDGF influences the activation of the NF-κB and MAPK pathways, western blotting was used to detect pathway-related protein phosphorylation. The expression of total and phosphorylated IKKα, P65, and IκBα was used to represent the activation of the NF-κB pathway (Fig. 5A–D). DSS induction significantly activated the NF-κB pathway, whereas MYDGF partially inhibited activation. Similarly, the expression of total and phosphorylated ERK, JNK, and P38 proteins was used to assess the activation of MAPK pathways (Fig. 5E–H). We found that MYDGF inhibited MAPK pathway activation partially. Considering the proteins of colonic tissues (whole colon) were used, immunofluorescence assays were also conducted to detect P-P65 and P-ERK (Fig. 5I, J), corroborating with the western blotting results. MYDGF significantly reduced the number of P-P65 or P-ERK-positive cells in the colon epithelium, as well as in the muscular layer.

## Discussion

MYDGF, or “c19orf10”, was initially misidentified as IL-25; however, the study was retracted [25]. A previous study found that c19orf10 was significantly concentrated in patient synovial fluid [26]. Consequently, the protein was reported to be produced by cultured fibroblast-like synovial cells and fibroblasts in synovial tissue [27]. However, the discoverer was not named due to the function of c19orf10 remaining unclear. c19orf10 has been recently reported to be a protein secreted by bone marrow cells that can protect and repair the heart following myocardial infarction. In the present study, the function of c19orf10 was confirmed; therefore, c19orf10 was named as “Myeloid-derived growth factor (i.e., MYDGF)” [9]. MYDGF cannot be assigned to any known protein-folding class based on its 3D structure [10]. According to recent studies, MYDGF promotes angiogenesis and inhibits inflammation [11, 12]. However, the effects of MYDGF on macrophages and inflammation remain unclear. Therefore, the authors investigated the effects of MYDGF on inflammation in a DSS-induced colitis model.

After feeding mice with 3% DSS in drinking water for 7 days, MYDGF ameliorated symptoms caused by DSS consumption, such as bloody and liquid stools, and weight loss, indicating that MYDGF treatment reduced the DAI score. After euthanizing the mice, the colons were collected for histological and apoptotic detection. MYDGF restored colon length and HAI scores and reduced colon apoptosis. Whole blood was collected for routine blood tests, which revealed that drinking of DSS-containing water reduced RBC, HCT, and HGB levels. However, MYDGF could only relieve the increased WBC and LYMPH that were caused by DSS consumption. Therefore, MYDGF may relieve DSS-induced colitis by inhibiting inflammation.

Considering its simplicity and high similarity to human UC, DSS colitis is often used to elucidate the molecular and cellular pathways involved in the pathogenesis of acute colitis [16, 17]. Although its etiology is unclear, UC is generally believed to be caused by the failure of the intestinal epithelial barrier, with the infiltration of inflammatory cells as the main pathological cause [3, 18]. DSS can disrupt the integrity of the intestinal epithelium, leading to a localized inflammatory response [16–18]. This present study examined the expression of inflammatory factors IL-6, TNF-α, and IL-1β in the serum of mice and confirmed that MYDGF can alleviate the inflammatory response. Since COX2 and iNOS are involved in inflammatory responses, COX2 and iNOS expression in colon tissue was also examined. The anti-inflammatory ability of MYDGF was further confirmed. The expression of intercellular cell adhesion molecules (such as Occludin and E-Cad) confirmed that MYDGF can maintain the integrity of colon epithelium. The results obtained from the analyses of the inflammatory factor TNF-α and anti-inflammatory factor IL-4 suggest that MYDGF may be involved in regulation of the inflammatory microenvironment.

Although MYDGF has a potentially therapeutic effect on DSS colitis, the mechanism by which it inhibits inflammation remains unclear. Macrophages are tissue sentinels, whose activation is pivotal for the restoration of innate immune responses and functioning of the main inflammatory response [28]. Targeting the differentiation of intestinal macrophages holds promise as a treatment for intestinal inflammatory diseases, such as IBD. Both M1 and M2 polarization of macrophages are known to be closely related to inflammatory responses [29, 30]. Therefore, the present study examined the polarization of macrophages in the colon tissue of mice. MYDGF reduced the number and proportion of M1 macrophages. Moreover, MYDGF increased the number and proportion of M2 macrophages.

DSS colitis activates the NF-κB and MAPK pathways [24], whereas kinsenoside regulates M1/M2 polarization of macrophages by inactivating the NF-κB and MAPK signaling pathways [31]. Inhibition of activated NF-κB and MAPK pathways is a potential mechanism for DSS colitis treatment [32–34]. Therefore, the authors examined the phosphorylation of key proteins involved in the NF-κB and MAPK pathways and found that MYDGF inhibited activation of the NF-κB and MAPK pathways. The results suggested that MYDGF may inhibit activation of the NF-κB and MAPK pathways to regulate intestinal macrophage polarization. Notably, MYDGF act on the macrophages within the colon epithelium, as well as the smooth muscle layer. The findings demonstrate that MYDGF has a wide range of functions. The effect and mechanism of MYDGF need to be studied further.

The results of the present study imply that MYDGF significantly alleviates DSS-induced colitis in mice and that MYDGF can reduce colon tissue apoptosis and inflammation caused by DSS water drinking and maintain the integrity of colon epithelium. In vivo, MYDGF has the potential to inhibit the M1 macrophage and promote the M2 macrophage, which could be attributed to MYDGF inhibiting the activation of the NF-κB and MAPK pathways.

## Materials and methods

### Animal model

Male C57BL/6 mice (8 weeks old) were provided by SPF Biotechnology Co. Ltd (Beijing, China). All procedures were approved by the Institutional Animal Care and Use Committee of Beijing Stomatological Hospital (Ethical code: No. KQYY-202210-001). Twenty-four mice were randomly separated into 4 groups (Ctrl group, MYDGF group, DSS group, and DSS+MYDGF group) after one week of adaptive feeding. Distilled water was used to configure DSS (9011-18-1, MP Biomedicals, CA, USA) into 3% solution as drinking water for the experimental groups. MYDGF protein (HY-P73303, MCE, NJ, USA) was diluted with distilled water to a concentration of 500 μg/mL. MYDGF and DSS+MYDGF groups were injected with MYDGF via tail vein on 1d, 3d, and 5d after the experiment began (5 μg/mouse). The same volume of distilled water was injected into the tail vein of DSS group.

The weight of the mice and the shape and consistency of stools were measured daily. Disease activity index (DAI) was used to evaluate the severity of colitis. Based on the references, DAI scores were assessed for each mouse includes weight loss, stool shape and bloody stool [35]. DAI rating from day 1 of DSS processing to day 7. On day 7, blood was collected from eyes after anesthesia, and all mice were sacrificed for cervical dislocation. The complete colon from the epityphlon to the anus was collected and the length of the colon was measured. Photographs of the colon were obtained immediately after the sample is collected.

### Blood routine examination

Mice blood were collected using blood collection vessels containing EDTA anticoagulant. White blood cells, red blood cells and other indicators were detected by automatic blood routine analyzer (DxH800, Beckman, CA, USA).

### Hematoxylin and eosin (H&E) staining and histological activity index (HAI)

Colon tissue samples were collected and fixed in 4% formalin for 48 h. After dehydration, waxdip and embedding, samples were sectioned at 5-µm thickness. Sections were rehydrated with graded ethanol and vitrified by dimethylbenzene. H&E staining was done to observe the histological changes. Five different views in each section were captured. The HAI was calculated based on mucosal inflammation and the degree of epithelial [35].

### Terminal deoxynucleotidyl transferase dUTP nick end labeling (TUNEL) assay

Paraffinized samples were sectioned at 5-μm thickness. TUNEL staining was performed with a One Step TUNEL Apoptosis Assay Kit (C1090, Beyotime, Shanghai, China) according to the manufacturer’s instructions. Following the TUNEL reaction, slides were treated with DAPI (F6057, Sigma, MO, USA). Images were captured under a confocal microscope and the number of TUNEL-positive cells was calculated in six random fields per slide.

### Immunohistochemistry (IHC) and immunofluorescence (IF) staining

Paraffin sections for IHC staining after dewaxing were antigen retrieval using microwave heating. Then, 3% Bovine Serum Albumin (BSA) were used to block the non-specific antigen. Sections were incubated with primer antibody (Caspase 3, A21677, Abclonal, Wuhan, China; E-Cad, A20798, Abclonal; COX2, A3560, Abclonal; Occludin, 27260-1-AP, Proteintech, Rosemont, USA; CD86, A1199, Abclonal; Arg1, 16001-1-AP, Proteintech; P-P65, AP1294, Abclonal; P-ERK, AP0886, Abclonal) overnight at 4 ℃. The next day, different second antibodies (PV-6001 and PV-60002, ZSGB-Bio, Beijing, China) were incubated for 1 h at 25 ℃. IHC was performed DAB kit (SP-9000, ZSGB-Bio). IF was used TSA amplification fluorescence kit (abs50012, Absin, Shanghai, China). Five different views in each section were captured. The integral optical density (IOD) and positive area/cells were calculated by ImageJ Pro Plus.

### Western blot

The colonic tissues were lysed in RIPA lysis buffer containing protease inhibitor. Proteins were extracted and quantified using BCA assay (P0011, Beyotime, China). The total protein of each sample was 15 μg. The primary antibody (Bax, 60267-1-Ig, Proteintech; Bcl-2, 68103-1-Ig, Proteintech; Caspase 3, A21677, Abclonal; E-Cad, A20798, Abclonal; COX2, A3560, Abclonal; Occludin, 27260-1-AP, Proteintech; iNOS, 18985-1-AP, Proteintech; TNF-α, 17590-1-AP, Proteintech; IL-4, 66142-1-Ig, Proteintech; CD80, 66406-1-Ig, Proteintech; CD68, 28058-1-AP, Proteintech; Arg-1, 16001-1-AP, Proteintech; P-IKKα, AP1066, Abclonal, IKKα, A19694, Abclonal; P-P65, AP1294, Abclonal; P65, 66535-1-Ig, Proteintech; P-IκBα, AP0999, Abclonal; IκBα, 10268-1-AP, Proteintech; P-ERK, AP0886, Abclonal; ERK, 11257-1-AP, Proteintech; P-JNK, 80024-1-RR, Proteintech; JNK, 24164-1-AP, Proteintech; P-P38, 28796-1-AP, Proteintech; P38, 14064-1-AP, Proteintech; β-action, AC026, Abclonal) were incubated overnight at 4 ℃ after proteins were transferred to Polyvinylidene Fluoride membrane. Next day, the membranes were incubated with different secondary antibodies (AS003 and AS014, Abclonal). Each primary antibody was incubated in the whole membrane. The experiment was repeated three times independently. The repeats were performed with lysates from different mice. Optical densities (OD) of blots were determined in the Image J software. The result is expressed as the ratio of the protein OD to the actin OD or other protein OD.

### Enzyme-linked immunosorbent assay (ELISA)

The levels of inflammatory cytokines IL-6, TNF-α and IL-1β were measured using ELISA kits as described [36]. The serum of mice was obtained by centrifugation of 1500 g and 15 min. ELISA was performed using commercial kits with antibodies against IL-6 (RK00008, Abclonal), TNF-α (430901, BioLegend, CA, USA) and IL-1β (432601, BioLegend). The results were assessed using a microplate reader at 450 nm and corrected at 570 nm.

### Statistical analysis

Results are presented as mean ± standard deviation (SD). All graphs were generated using GraphPad Prism 9.3 software (GraphPad Software, San Diego, USA). All data were analyzed using SPSS 22.0. Two-way analysis of variance (ANOVA) was used to compare the differences among two groups with multiple columns such as weight and DAI. One-way analysis of variance (ANOVA) was used to compare the differences among multiple groups and Bonferroni’s multiple comparisons test was used for post-hoc analysis of the ANOVA results. The unpaired non-parametric t-test was used to compare two groups for western blot. P-values < 0.05 were considered statistically significant.

### Supplementary Information

Below is the link to the electronic supplementary material.

Supplementary file1 (TIFF 17100 MB)

Supplementary file2 (TIFF 17100 MB)

Supplementary file3 (DOCX 16.3 KB)

## Data Availability

Authors confirm that all relevant data supporting the findings of this study are included in the article.
