# Supplementary figures and images for: Myeloid-derived growth factor ameliorates dextran sodium sulfate-induced colitis by regulating macrophage polarization
Source: J Mol Med (Berl). 2024 May 2;102(7):875–86. doi: 10.1007/s00109-024-02447-3 (PMC11213757; doi:10.1007/s00109-024-02447-3)

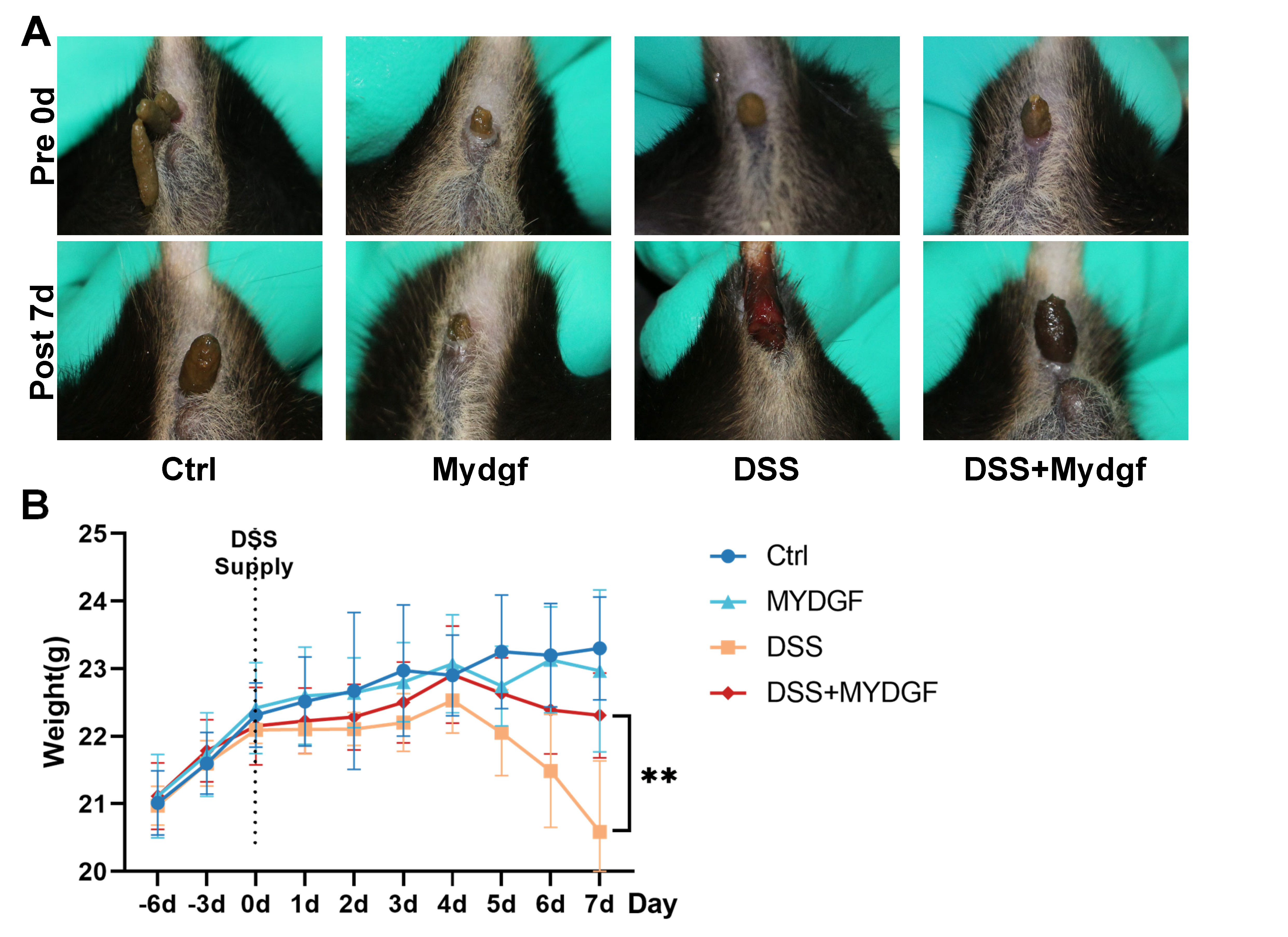

Supplement: Supplementary file 1 — Supplementary file1 (TIFF 17100 MB) [file 109_2024_2447_MOESM1_ESM.tiff]

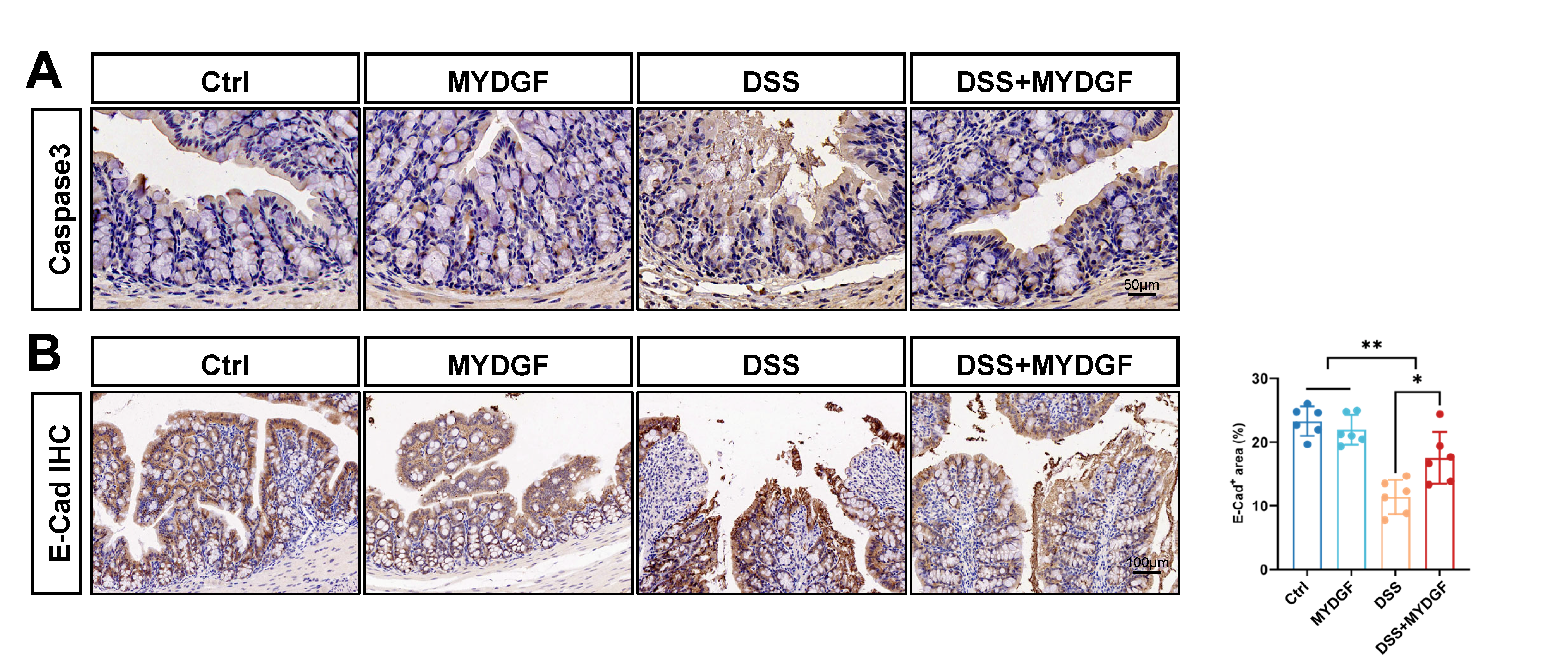

Supplement: Supplementary file 2 — Supplementary file2 (TIFF 17100 MB) [file 109_2024_2447_MOESM2_ESM.tiff]
