# Supplementary material for: Myeloid-derived growth factor ameliorates dextran sodium sulfate-induced colitis by regulating macrophage polarization
Source: J Mol Med (Berl). 2024 May 2;102(7):875–86. doi: 10.1007/s00109-024-02447-3 (PMC11213757; doi:10.1007/s00109-024-02447-3)
Supplement: Supplementary file 3 — Supplementary file3 (DOCX 16.3 KB) [file 109_2024_2447_MOESM3_ESM.docx]

| **Supplementary Table S1. Blood routine examination** | | | | |
| --- | --- | --- | --- | --- |
|  | Ctrl | Mydgf | DSS+H_2_O | DSS+Mydgf |
| MCV | 47.30±0.45 | 48.48±2.65 | 49.65±3.16 | 51.90±4.04 |
| MCH | 15.15±0.18 | 15.15±0.16 | 15.38±0.35 | 15.38±0.28 |
| MCHC | 320.67±3.67 | 313.33±16.62 | 310.50±16.57 | 297.33±19.43 |
| MPV | 6.87±0.16 | 6.87±0.17 | 6.92±0.19 | 6.95±0.21 |
| PCT | 0.64±0.04 | 0.64±0.06 | 0.57±0.10 | 0.64±0.06 |
| MONO% | 3.67±1.63 | 2.75±1.63 | 1.72±0.52 | 1.73±0.24 |
| MONO | 0.18±0.10 | 0.18±0.25 | 0.15±0.07 | 0.09±0.03 |
